# Beyond Net Ultrafiltration Rate: A Precision Fluid Management Paradigm Integrating Three Key Parameters for Pediatric Continuous Renal Replacement Therapy

**DOI:** 10.1097/CCE.0000000000001424

**Published:** 2026-05-25

**Authors:** Wenjun Liu, Chunxiao Wang, Changyang Ye, Xiaowei Xiong, Dong Li, Ke Bai, Chengjun Liu

**Affiliations:** All authors: Department of Critical Care Medicine, Children’s Hospital of Chongqing Medical University, National Clinical Research Center for Children and Adolescents’ Health and Diseases, Chongqing Municipal Health Commission Key Laboratory of Children’s Vital Organ Development and Diseases, Chongqing, China.

**Keywords:** continuous renal replacement therapy, fluid management, fluid overload clearance percentage, net ultrafiltration rate, pediatrics

## Abstract

**IMPORTANCE:**

Fluid management in critically ill children receiving continuous renal replacement therapy (CRRT) is challenging due to limitations of conventional metrics. Net ultrafiltration rate (NUFR, mL/kg/hr) lacks pediatric specificity, whereas net fluid balance (NFB, mL/kg) is confounded by ongoing fluid inputs.

**OBJECTIVES:**

To introduce and validate fluid overload clearance percentage (FOCP, %)—a novel metric expressing NFB as a proportion of initial fluid overload—and to establish a tri-parameter framework integrating NUFR, NFB, and FOCP for precision fluid management.

**DESIGN, SETTING, AND PARTICIPANTS:**

Single-center retrospective cohort study in a tertiary PICU. All children receiving CRRT between 2012 and 2023 with greater than or equal to 24 hours of therapy were included (*N* = 432).

**MAIN OUTCOMES AND MEASURES:**

First 24-hour NUFR, NFB, and FOCP were analyzed. To illustrate the relationship between prescription intensity and achieved fluid removal, these parameters were compared between survivors and nonsurvivors. Subsequently, to identify physiologic determinants independent of mortality, survivors were stratified by body weight, baseline fluid overload (FO), and capillary leak index.

**RESULTS:**

Nonsurvivors received higher NUFR (median 4.8 mL/kg/hr [interquartile range 2.9–7.7] vs. 3.2 mL/kg/hr [1.6–5.6], *p* < 0.001) yet achieved comparable NFB (–22.6 mL/kg [–56.9 to 12.0] vs. –18.6 mL/kg [–48.2 to 2.3], *p* = 0.849) and FOCP (–41.0% [–102.7 to 21.9] vs. –44.2% [–128.1 to 17.3], *p* = 0.350). Body weight was the dominant determinant of NUFR (*r* = –0.548, *p* < 0.001). In contrast, FOCP demonstrated no significant correlation with illness severity (Pediatric Risk of Mortality-III, *r* = –0.012, *p* = 0.808) or baseline FO (*r* = –0.067, *p* = 0.165), and only a weak correlation with body weight (*r* = 0.279, *p* < 0.001).

**CONCLUSIONS AND RELEVANCE:**

We propose a tri-parameter precision management framework: NUFR serves as the weight-adjusted prescription parameter, NFB as the systemic safety outcome, and FOCP as the individualized clearance target. This integrated approach enables goal-directed therapy tailored to each patient’s physiology in pediatric CRRT.

**KEY POINTS**

**Question**: Given the physiologic complexity of pediatric continuous renal replacement therapy (CRRT), can a single parameter (net ultrafiltration rate [NUFR]) adequately guide therapy, or does a tri-parameter framework integrating NUFR, net fluid balance (NFB), and fluid overload clearance percentage (FOCP) enable more individualized care?

**Findings**: In this cohort of 432 children, nonsurvivors received higher NUFR yet achieved similar NFB and FOCP as survivors due to higher concurrent fluid inputs. NUFR was strongly weight-dependent even after weight-indexing (mL/kg/hr), because baseline maintenance fluid requirements in children scale inversely with body weight—indicating that a single universal NUFR target is physiologically untenable across the pediatric weight spectrum, whereas FOCP remained stable across weight, illness severity, and fluid overload strata.

**Meanings**: A tri-parameter framework integrating NUFR (prescription), NFB (safety), and FOCP (individualized target) enables goal-directed, physiology-based fluid management in pediatric CRRT.

Fluid overload (FO) at continuous renal replacement therapy (CRRT) initiation is a dose-dependent predictor of mortality in critically ill children (1, 2). This risk, however, presents a clinical challenge: despite the control CRRT affords over fluid removal, uncertainty persists regarding the optimal metric to guide its prescription, particularly in anuric children whose fluid homeostasis is entirely CRRT-dependent.

The net ultrafiltration rate (NUFR, mL/kg/hr ), constitutes the conventional cornerstone of CRRT dosing. In adult critical care, evidence consistently demonstrates a U-shaped relationship between NUFR and survival, yielding evidence-based targets typically ranging from 1.0 to 1.75 mL/kg/hr (3–5). The direct extrapolation of these adult-derived targets to pediatric practice, however, is physiologically problematic. The Holliday-Segar formula, which is commonly used to estimate pediatric maintenance fluid requirements, establishes an inverse relationship between body weight and weight-adjusted hourly fluid needs: a 10 kg infant has a full maintenance requirement of approximately 4 mL/kg/hr. In the context of CRRT, where nutritional support need not be killed for fluid control, this baseline rate—which already exceeds adult-derived safety limits (1.0–1.75 mL/kg/hr)—illustrates the physiologic challenge of applying adult NUFR targets to children (6).

Despite this physiologic imperative, pediatric literature on NUFR dosing remains limited. Frequently cited proposals suggest a uniform range of 0.5–2 mL/kg/hr (7–9). These recommendations are largely derived from mean prescribed rates reported in small, heterogeneous pediatric cohorts, without stratification by body weight or systematic evaluation of the relationship between prescribed dose and outcomes. This uniform approach risks under-treating infants while potentially harming larger adolescents.

The path from prescription (NUFR) to systemic outcome is further complicated by net fluid balance (NFB, mL/kg). Therapeutically, a greater degree of FO necessitates both a higher NUFR and a more negative NFB. However, NFB—while intuitively appealing—is confounded because it does not isolate the effect of CRRT. A negative NFB can result from either effective ultrafiltration or high native urine output, whereas a positive NFB can occur despite aggressive ultrafiltration if concurrent fluid inputs (medications, nutrition, blood products) are substantial (10, 11). This composite nature limits NFB as a specific gauge of CRRT efficacy. The common clinical observation of a dissociation between a high prescribed NUFR and a less negative NFB underscores this limitation: substantial fluid administration can offset aggressive ultrafiltration (“competing flows”), whereas significant native urine output may yield a negative NFB despite a modest NUFR.

Underpinning this complexity is the role of endothelial dysfunction and capillary leak syndrome. This pathology can manifest as significant FO, yet it creates a paradox: the intravascular compartment may exist in a state of effective hypovolemia despite total body fluid excess. Consequently, for any given level of FO, significant capillary leak narrows the therapeutic window for fluid removal, lowering hemodynamic tolerance to both NUFR and negative NFB. In this study, we employ the capillary leak index (CLI) as a practical marker to estimate its severity (12, 13).

The intricate interplay among body weight (dictating baseline fluid requirements), fluid overload (FO) (defining the therapeutic goal), and capillary leak (constraining hemodynamic tolerance)—each exerting independent and sometimes opposing influences on fluid management—underscores the need for a unified, patient-centered metric that contextualizes therapeutic effort against the individual’s specific physiologic burden.

This study introduces and validates the fluid overload clearance percentage (FOCP), a novel metric defined as:


$$FOCP\;(\%)=\frac{\left[Net\;fluid\;balance\;at\;24\;hr\;(mL/kg)\right]}{\left[FO\;at\;CRRT\;initiation\;(\%)\times \;10\right]}\times 100\%$$

where the number of FO% × 10 approximates the excess volume in mL/kg (e.g., 5% FO corresponds to 50 mL/kg). By reframing systemic fluid removal relative to the initial problem’s scale, FOCP may serve as a cornerstone for individualized, goal-directed fluid management. We hypothesized that FOCP would demonstrate consistent properties across diverse physiologic strata—unlike NUFR, which is known to be weight-dependent, and NFB, which is confounded by concurrent inputs. To test this hypothesis, we analyzed first 24-hour NUFR, NFB, and FOCP in a large pediatric CRRT cohort, first examining their relationship with mortality and then exploring their physiologic determinants through stratified analyses. Based on these findings, we aimed to establish a physiologically grounded framework integrating all three parameters for precision fluid management.

## METHODS

### Study Design and Population

This single-center, retrospective cohort study was conducted in a tertiary academic PICU. We screened all consecutive patients from the neonatal period to 18 years old who received CRRT between January 1, 2012, and December 31, 2023. This study was reviewed and approved as human subject research by the Ethics Committee of the Children’s Hospital Affiliated to Chongqing Medical University (File No. 2023(503)) on November 20, 2023. All procedures were performed in accordance with the committee’s ethical standards and the Declaration of Helsinki (1975). Informed consent was waived by the committee due to the retrospective, low-risk design of the study, and a formal institutional review board (IRB) supplementary statement confirming this waiver is provided as **IRB Supplementary Document** (https://links.lww.com/CCX/B631).

**Figure 1.**
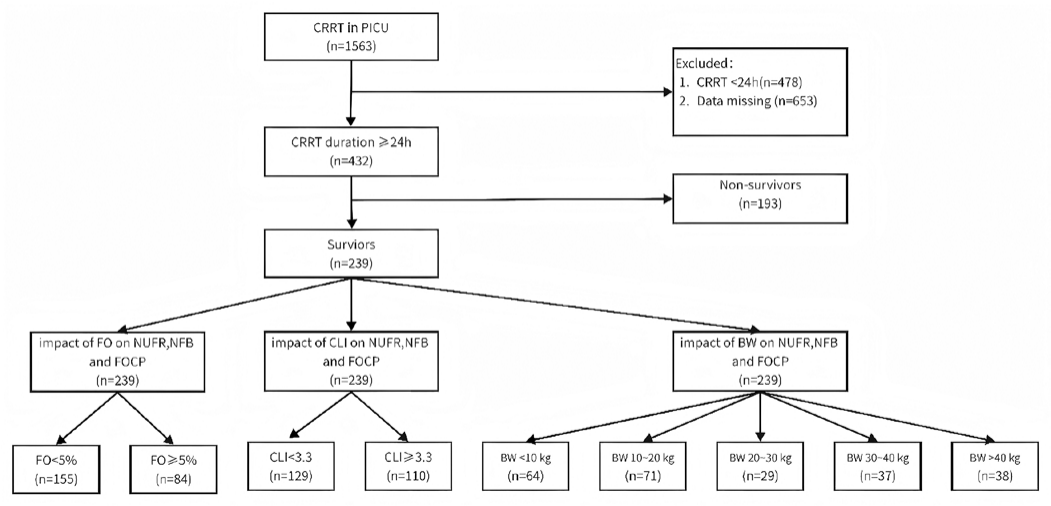
Patient flow diagram. The flowchart illustrates the inclusion and exclusion process for the retrospective cohort study of critically ill children receiving continuous renal replacement therapy (CRRT) between 2012 and 2023. A total of 432 patients met the inclusion criteria and constituted the final study population for the comparative analysis of fluid management metrics. Key exclusion criteria were CRRT duration of less than 24 hours and missing essential data for calculating fluid overload percentage or net ultrafiltration volume. BW = body weight, CLI = capillary leak index, FO = fluid overload, FOCP = fluid overload clearance percentage, NFB = net fluid balance, NUFR = net ultrafiltration rate.

Patient selection is detailed in **Figure 1**. Inclusion required CRRT initiation for any indication. Exclusion criteria comprised: 1) CRRT duration less than 24 hours, and 2) missing essential data for calculating key variables (FO, NUF volume). To elucidate physiologic relationships independent of mortality, all analyses examining the impact of body weight, FO, and CLI were performed within the 28-day survivor cohort.

All fluid management metrics were calculated for the first 24 hours of CRRT. In our cohort, a substantial proportion of patients did not remain on CRRT beyond 48–72 hours; therefore, the 24-hour window was chosen to maximize cohort inclusiveness.

### Data Collection and Variable Definitions

Demographic, clinical, and laboratory data were extracted from electronic medical records. All fluid management metrics and hemodynamic parameters were calculated for the first 24 hours of CRRT to standardize comparison. All weight-based metrics (FO, NUFR, NFB, fluid intake) were indexed to baseline weight, defined as body weight measured at CRRT initiation, to avoid confounding by fluid accumulation.

#### Fluid Overload (FO):

Calculated at CRRT initiation: FO (%) = [(Cumulative Fluid Intake (L) – Cumulative Fluid Output (L))/Baseline Weight (kg)] × 100% (14)

#### Capillary Leak Index (CLI):

Calculated as [C-reactive protein (mg/dL)/serum albumin (g/L)] × 100, using values obtained within 24 hours before CRRT initiation, representing the baseline inflammatory/endothelial state at therapy onset (12, 13).

#### Net Ultrafiltration Volume (NUFV):

NUFV (mL) = Total volume of fluid removed via the CRRT circuit – (Replacement fluids + Dialysate + Return-line flush volumes) over the 24-hour period.

#### Net Ultrafiltration Rate (NUFR):

NUFR (mL/kg/hr) = Total Net Ultrafiltration Volume (mL)/[Baseline Weight (kg) × CRRT Time (h)].

#### Net Fluid Balance (NFB):

NFB (mL/kg) over the first 24 hours of CRRT was calculated as: NFB (mL/kg) = [Total Fluid Intake volume (mL) – Total Fluid Output volume (mL) – Total NUFV (mL)]/Baseline Weight (kg).

#### Fluid Overload Clearance Percentage (FOCP):

FOCP (%) = [Net Fluid Balance at 24 hours (mL/kg)]/[FO at CRRT initiation (%) × 10] × 100%. This calculation expresses the 24-hour NFB as a proportion of the initial FO volume, where FO% × 10 approximates the excess volume in mL/kg.

#### Vasoactive-inotropic score (VIS):

Calculated as [dopamine + dobutamine + 100 × epinephrine + 100 × norepinephrine + 10 × milrinone] (all doses in μg/kg/min) + 10,000 × vasopressin (U/kg/min).

#### Hemodynamic Assessment:

Assessed by the change in mean arterial pressure (ΔMAP) and the change in Vasoactive-Inotropic Score (ΔVIS) from CRRT initiation to 24 hours.

### Subgroup Analysis

To dissect the independent relationships of key physiologic variables with fluid management parameters, we performed an a priori stratification of survivors based on:

- Body Weight (< 10 kg, 10–< 20 kg, 20–< 30 kg, 30–< 40 kg, ≥ 40 kg)
- Baseline fluid overload (< 5% vs. ≥ 5%) (15)
- Baseline CLI (< 3.3 vs. ≥ 3.3) (13)

### Statistical Analysis

Continuous data are presented as median (interquartile range) and compared using the Mann-Whitney *U* or Kruskal-Wallis test. Categorical data are presented as *n* (%) and compared using the chi-square test. Spearman correlation was used for association analysis. A two-sided *p* value of less than 0.05 was considered significant. Analyses were performed using R software (Version 4.3.0; R Foundation for Statistical Computing, Vienna, Austria).

## RESULTS

### Cohort Characteristics and Prescription-Outcome Comparison

The final cohort included 432 critically ill children (Fig. 1). As summarized in **Table 1**, nonsurvivors (*n* = 193) presented with greater physiologic derangement at baseline than survivors (*n* = 239). They were younger (median 21 vs. 58 mo, *p* < 0.001), had lower body weight (10 vs. 15 kg, *p* < 0.001), higher illness severity (Pediatric Risk of Mortality [PRISM]-III score 14 vs. 8, *p* < 0.001), greater fluid overload (FO 6.1% vs. 2.7%, *p* < 0.001), and a higher CLI (3.8 vs. 3.1, *p* = 0.001). Nonsurvivors received more intensive CRRT than survivors. They had higher blood flow rates (3.6 vs. 3.3 mL/kg/min, *p* = 0.022) and higher total effluent rates (49.6 vs. 41.4 mL/kg/hr, *p* < 0.001). Consequently, their net ultrafiltration rate was higher (NUFR: median 4.8 mL/kg/hr [interquartile range 2.9–7.7] vs. 3.2 mL/kg/hr [1.6–5.6], *p* < 0.001). Despite this intensified prescription, the achieved NFB was similar between groups (NFB: –22.6 mL/kg [–56.9 to 12.0] vs. –18.6 mL/kg [–48.2 to 2.3], *p* = 0.849), as was the FOCP (–41.0% [–102.7 to –21.9] vs. –44.2% [–128.1 to –17.3], *p* = 0.350). Nonsurvivors had significantly higher concurrent fluid inputs, including total fluid intake (5.4 vs. 3.8 mL/kg/hr, *p* < 0.001), RBC transfusion, and albumin infusion (both *p* < 0.001). Hemodynamic changes during the first 24 hours did not differ between groups. ΔMAP was 1.0 mm Hg in both groups (*p* = 0.507), and ΔVIS was 0.0 in both groups (*p* = 0.017), indicating minimal clinically relevant differences.

**TABLE 1.** Baseline Characteristics, Continuous Renal Replacement Therapy Parameters, and Clinical Outcomes of the Study Population

Characteristic	Survivors (*n* = 239)	Nonsurvivors (*n* = 193)	*p*
Demographics			
Age, mo	58.0 (12.0 to 132.0)	21.0 (3.0 to 102.0)	< 0.001
Male sex, *n* (%)	137 (57.3)	108 (56.0)	0.776
Body weight, kg	15.0 (9.0 to 34.0)	10.0 (5.0 to 25.0)	< 0.001
Pediatric Risk of Mortality-III score	8.0 (5.0 to 10.0)	14.0 (10.0 to 18.0)	< 0.001
Capillary leak index	3.1 (2.4 to 9.0)	3.8 (2.7 to 16.2)	0.001
Fluid overload at CRRT initiation, %	2.7 (0.7 to 7.0)	6.1 (2.1 to 10.3)	< 0.001
CRRT parameters (24 hr)			
QB/BW, mL/kg/min	3.3 (2.5 to 4.0)	3.6 (2.9 to 4.3)	0.022
(Qd+Qf)/BW, mL/kg/hr	41.4 (30.8 to 58.2)	49.6 (35.6 to 63.3)	< 0.001
Net ultrafiltration rate, mL/kg/hr	3.2 (1.6 to 5.6)	4.8 (2.9 to 7.7)	< 0.001
Net fluid balance, mL/kg	–18.6 (–48.2 to 2.3)	–22.6 (–56.9 to 12.0)	0.849
Fluid overload clearance percentage, %	–44.2 (–128.1 to –17.3)	–41.0 (–102.7 to –21.9)	0.350
Fluid and blood product input (24 hr)			
Total fluid intake, mL/kg/hr	3.8 (2.6 to 5.3)	5.4 (3.7 to 7.7)	< 0.001
RBC transfusion, ×10^−2^ U/kg	0.0 (0.0 to 3.3)	0.0 (0.0 to 7.4)	< 0.001
Albumin infusion, g/kg	0.4 (0.0 to 1.3)	1.1 (0.3 to 2.9)	< 0.001
Hemodynamic changes (24 hr)			
Change in mean arterial pressure, mm Hg	1.0 (–9.0 to 11.0)	1.0 (–6.0 to 9.0)	0.507
Change in vasoactive-inotropic score	0.0 (0.0 to 0.0)	0.0 (0.0 to 4.5)	0.017
Outcomes			
ICU length of stay, d	10.0 (6.0 to 17.0)	9.0 (5.0 to 16.5)	0.049
Duration of mechanical ventilation, d	4.8 (0.0 to 9.7)	7.0 (3.0 to 13.0)	< 0.001
Hospitalization duration, d	25.0 (18.0 to 35.0)	16.0 (7.0 to 31.5)	< 0.001

CRRT = continuous renal replacement therapy, QB = blood flow, Qd = dialysate flow, Qf = replacement fluid flow.

Data are presented as median (interquartile range) or *n* (%). Nonsurvivors were younger, had lower body weight, and greater illness severity (Pediatric Risk of Mortality-III score) at baseline. They also presented with significantly higher fluid overload (FO) and capillary leak index (CLI) at CRRT initiation. Despite receiving more intensive CRRT, as evidenced by higher blood flow rate (QB/BW), total effluent rate ((Qd+Qf)/BW), and net ultrafiltration rate, nonsurvivors achieved a similar net fluid balance and FO clearance percentage compared with survivors.

### Stratified Physiologic Analysis of Survivors

To identify the physiologic determinants of each fluid management parameter independent of mortality, we performed stratified analyses within the survivor cohort (*n = 239*), examining the effects of body weight, baseline FO, and CLI (**Table 2**).

**TABLE 2.** Stratified Analysis of Survivors by Body Weight, Baseline Fluid Overload, and Capillary Leak Index

Stratification	Subgroup	*n*	Body Weight (kg)	Fluid Overload (%)	Net Ultrafiltration Rate (mL/kg/hr)	Net Fluid Balance (mL/kg)	Fluid Overload Clearance Percentage (%)
Body weight	< 10 kg	64	6.4 (3.5 to 7.8)	5.6 (2.0 to 11.3)	5.3 (3.4 to 7.1)	–36.2 (–72.5 to –8.2)	–54.1 (–90.2 to –8.6)
	10 to < 20 kg	71	12.5 (11.0 to 15.0)	3.2 (0.6 to 7.9)	4.0 (2.6 to 6.0)	–27.0 (–51.9 to 5.9)	–50.1 (–110.7 to 23.0)
	20 to < 30 kg	29	23.0 (20.0 to 27.0)	2.1 (0.8 to 4.9)	2.2 (0.9 to 4.2)	–15.8 (–48.4 to 4.5)	–52.9 (–180.3 to –5.5)
	30 to < 40 kg	37	35.0 (31.5 to 36.3)	1.5 (0.5 to 6.0)	2.5 (1.3 to 3.9)	–12.7 (–32.0 to 5.2)	–31.0 (–342.5 to 33.1)
	≥ 40 kg	38	50.0 (45.0 to 61.3)	1.0 (0.2 to 2.8)	1.5 (0.9 to 2.5)	–6.6 (–24.9 to 9.2)	–20.8 (–162.8 to 59.0)
	*p*		< 0.001	< 0.001	< 0.001	0.010	0.576
Fluid overload	< 5%	155	20.0 (11.0 to 37.0)	1.2 (0.4 to 2.5)	2.7 (1.3 to 4.3)	–11.2 (–35.9 to 8.7)	–52.9 (–227.9 to 66.5)
	≥ 5%	84	10.8 (7.0 to 20.0)	8.6 (6.5 to 11.7)	4.5 (3.1 to 6.6)	–37.3 (–72.7 to –12.2)	–42.7 (–70.8 to –10.9)
	*p*		< 0.001	< 0.001	< 0.001	< 0.001	0.436
Capillary leak index	< 3.3	129	14.0 (7.8 to 30.0)	2.0 (0.5 to 5.5)	2.9 (1.3 to 5.1)	–13.1 (–39.2 to 5.4)	–39.9 (–124.4 to 26.3)
	≥ 3.3	110	16.5 (9.0 to 33.5)	4.1 (1.5 to 8.4)	3.9 (2.1 to 5.9)	–29.2 (–52.5 to –0.2)	–50.8 (–137.2 to 10.1)
	*p*		0.926	< 0.001	0.006	0.013	0.419

Data are presented as median (interquartile range). Lower body weight was strongly associated with higher baseline fluid overload (FO), higher prescribed net ultrafiltration rate (NUFR), and a more negative net fluid balance (NFB) (all *p* < 0.001, Kruskal-Wallis test). In contrast, the fluid overload clearance percentage (FOCP) remained consistent across all body weight strata (*p* = 0.576). Patients with FO ≥ 5% received higher NUFRs to achieve more negative NFB, yet their FOCP was comparable to the FO < 5% group.

Body weight was strongly associated with prescribed NUFR (*p* < 0.001). Infants less than 10 kg received a median NUFR of 5.3 mL/kg/hr [3.4–7.1], compared with 1.5 mL/kg/hr [0.9–2.5] in children greater than or equal to 40 kg. NFB was also more negative in smaller children (*p* = 0.010). In contrast to both NUFR and NFB, FOCP remained consistent across all weight strata (*p* = 0.576), ranging from –54.1% [–90.2 to –8.6] in infants to –20.8% [–162.8 to 59.0] in adolescents.

Patients with baseline FO greater than or equal to 5% received higher NUFR than those with FO less than 5% (4.5 mL/kg/hr [3.1–6.6] vs. 2.7 mL/kg/hr [1.3–4.3], *p* < 0.001) and achieved more negative NFB (–37.3 mL/kg [–72.7 to –12.2] vs. –11.2 mL/kg [–35.9 to 8.7], *p* < 0.001). However, their FOCP was comparable to the less overloaded group (–42.7% [–70.8 to –10.9] vs. –52.9% [–227.9 to 66.5], *p* = 0.436).

Patients with CLI greater than or equal to 3.3 similarly received higher NUFR than those with CLI less than 3.3 (3.9 mL/kg/hr [2.1–5.9] vs. 2.9 mL/kg/hr [1.3–5.1], *p* = 0.006) and achieved more negative NFB (–29.2 mL/kg [–52.5 to –0.2] vs. –13.1 mL/kg [–39.2 to 5.4], *p* = 0.013). Again, FOCP showed no difference between groups (–50.8% [–137.2 to 10.1] vs. –39.9% [–124.4 to 26.3], *p* = 0.419).

**Figure 2.**
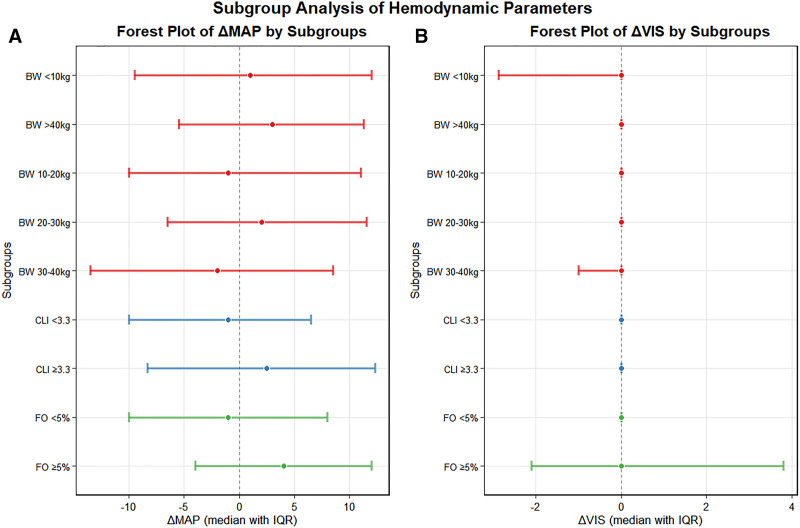
Hemodynamic stability during the first 24 hours of continuous renal replacement therapy (CRRT). *Forest plots* display median values (*symbols*) with interquartile ranges (*horizontal lines*) for changes in (**A**) vasoactive-inotropic score (ΔVIS) and (**B**) mean arterial pressure (ΔMAP) after 24 hours of CRRT, stratified by body weight (BW), baseline fluid overload (FO), and capillary leak index (CLI). The universal hemodynamic stability is demonstrated by two key findings: 1) ΔVIS values are tightly distributed around zero across all patient subgroups, indicating no systematic need for increased vasopressor support, and 2) ΔMAP values remain within a narrow, clinically acceptable range. Notably, the FO greater than or equal to 5% and CLI greater than or equal to 5 subgroups show a positive ΔMAP trend, consistent with improved perfusion pressure after fluid removal in these cohorts. The *vertical dashed line* indicates the zero-change reference.

Hemodynamically, fluid removal was well-tolerated across all subgroups. Both the FO greater than or equal to 5% and CLI greater than or equal to 3.3 subgroups exhibited a modest increase in mean arterial pressure (ΔMAP +4.0 mm Hg and +2.5 mm Hg, respectively, both *p* < 0.05), whereas vasopressor support (ΔVIS) remained stable (all *p* > 0.05). The hemodynamic stability across different subgroups is visually summarized in **Fig. 2** and **Table S1** (https://links.lww.com/CCX/B631).

### Correlational Analysis

To further characterize the relationships between patient characteristics and fluid management parameters, we performed correlational analysis (**Table 3**). NUFR demonstrated a strong negative correlation with body weight (*r* = –0.548, *p* < 0.001) and a strong positive correlation with baseline FO (*r* = 0.455, *p* < 0.001). NFB showed moderate correlations with multiple variables. In contrast, FOCP was largely independent of potential confounders: it showed no significant correlation with illness severity (PRISM-III, *r* = –0.012, *p* = 0.808) or baseline FO (*r* = –0.067, *p* = 0.165), and only a weak correlation with body weight (*r* = 0.279, *p* < 0.001).

**TABLE 3.** Correlations Among Fluid Management Parameters and Clinical Variables in Survivors

Variable	Pediatric Risk of Mortality-III	Body Weight	Fluid Overload	Capillary Leak Index	Net Fluid Balance	Change in Mean Arterial Pressure	Change in Vasoactive-Inotropic Score
Net ultrafiltration rate (mL/kg/hr)	*r* = 0.200 *p* = 0.002	*r* = –0.548 *p* < 0.001	*r* = 0.455 *p* < 0.001	*r* = 0.130 *p = 0.045*	*r* = –0.657 *p* < 0.001	*r* = 0.063 *p = 0.333*	*r* = 0.150 *p = 0.821*
Net fluid balance (mL/kg)	*r* = –0.083 *p* = 0.199	*r* = 0.277 *p* < 0.001	*r* = –0.311 *p* < 0.001	*r* = –0.154 *p* = 0.017	-	*r* = –0.093 *p* = 0.154	*r* = 0.005 *p* = 0.942
Fluid overload clearance percentage (%)	*r* = –0.012 *p* = 0.808	*r* = 0.279 *p* < 0.001	*r* = –0.067 *p* = 0.165	*r* = –0.115 *p* = 0.017	*r* = 0.827 *p* < 0.001	*r* = –0.101 *p* = 0.035	*r* = 0.099 *p* = 0.041

Table displays Spearman correlation coefficients (*r*) and corresponding *p* values. The net ultrafiltration rate (NUFR) demonstrated a strong negative correlation with body weight (*r* = –0.548, *p* < 0.001), establishing weight as the primary determinant of prescription intensity. NUFR was also strongly negatively correlated with net fluid balance (NFB) (*r* = –0.657, *p* < 0.001). In contrast, the fluid overload clearance percentage showed a very strong positive correlation with NFB (*r* = 0.827, *p* < 0.001) but was largely independent of illness severity (Pediatric Risk of Mortality-III) and baseline fluid overload, supporting its role as a potential universal efficiency metric.

## DISCUSSION

This study introduces and validates a physiologically grounded framework for precision fluid management in pediatric CRRT. By deconstructing the distinct roles of NUFR, NFB, and FOCP, we move beyond the limitations of single-parameter approaches to propose an integrated, goal-directed strategy. This work offers three novel contributions: 1) introduction of FOCP as a proportional efficiency metric that contextualizes fluid removal relative to initial overload, 2) demonstration that FOCP, unlike NUFR and NFB, is stable across weight and illness severity, and 3) integration of all three parameters into a dynamic, iterative clinical framework.

### Prescription-Outcome Decoupling and the Need for Multiple Parameters

Our analysis first reveals a dissociation between therapeutic intensity and achieved effect. Nonsurvivors received significantly higher NUFR yet achieved comparable NFB and FOCP. This finding is explained by substantially higher concurrent fluid inputs in nonsurvivors (Table 1), which offset aggressive ultrafiltration. The decoupling between what is prescribed (NUFR) and what is achieved (NFB, FOCP) underscores a fundamental limitation of relying on NUFR alone and motivates the need for a multi-parameter framework that captures both intensity and efficiency. This observation aligns with previous reports that fluid accumulation from ongoing resuscitation can negate the effects of even aggressive ultrafiltration in the sickest patients (16).

### Comparison With Existing Evidence

The strong weight-dependence of NUFR in children (*r* = –0.548, *p* < 0.001) stands in marked contrast to the relatively uniform targets established for adults (1.0–1.75 mL/kg/hr) (3–5) . This difference reflects the fundamental physiologic principle that maintenance fluid requirements scale inversely with body weight—a factor that is negligible in the narrow weight range of adults (typically 50–100 kg) but becomes dominant across the 30-fold weight spectrum of pediatric practice (from 3 kg infants to 100 kg adolescents).

Previous pediatric studies have reported mean NUFR values ranging from 0.5 to 2 mL/kg/hr across heterogeneous cohorts (7–9). Our stratified analysis extends these observations by demonstrating that this wide range is not primarily due to inter-study variability in clinical practice, but reflects the weight composition of the cohorts studied. Studies with predominantly infants reported rates at the upper end of this range; those with predominantly adolescents reported rates at the lower end. When stratified by body weight, NUFR shows a consistent weight-dependent pattern: infants in our cohort required a median of 5.3 mL/kg/hr, compared with 1.5 mL/kg/hr in adolescents. This finding explains why single-number recommendations have proven inadequate and supports the need for weight-stratified guidelines.

The observation that patients with higher baseline FO received more intensive CRRT is consistent with clinical intuition and prior recommendations (1). However, our finding that FOCP remained comparable regardless of FO severity adds a novel dimension: clinicians appear to titrate therapy to achieve a similar proportional reduction in fluid burden, even when the absolute amount to be removed varies substantially. This aligns with the concept of “proportional goals” increasingly recognized in other areas of critical care, where treatment is titrated to achieve a physiologic target relative to the patient’s baseline status rather than an absolute value (17).

### Physiologic Determinants of Each Parameter

Stratified analysis of survivors revealed distinct physiologic influences on each metric. NUFR was strongly weight-dependent in children, confirming that a single uniform target—such as those established for adults—is physiologically untenable in the pediatric population. NFB correlated with multiple factors, reflecting its composite nature as a summary of all fluid gains and losses. In contrast, FOCP remained stable across all weight, FO, and CLI strata, and showed no correlation with illness severity —a property that is clinically important as it positions FOCP as a physiology-independent efficiency metric that does not require adjustment for acute illness acuity, enabling a universal proportional goal across heterogeneously ill populations.

This proportional approach fundamentally differs from conventional absolute targets (e.g., –500 mL/24 hr) or weight-indexed rates (e.g., 2 mL/kg/hr), both of which fail to account for the initial fluid burden. FOCP answers a clinically more relevant question: “What fraction of the patient’s excess fluid has been removed?”—a metric that remains interpretable regardless of patient size or initial overload severity.

The weight-independence and stability of FOCP are clinically important because they indicate that it can serve as a universal efficiency metric—a proportional target that does not require adjustment for patient size or disease severity. This property allows clinicians to set consistent proportional goals across the entire pediatric spectrum while individualizing the NUFR prescription needed to achieve that goal. These findings have direct implications for clinical guidelines: rather than recommending a single NUFR range, future guidelines should provide weight-stratified targets, acknowledging that infants require rates at the upper end of the spectrum while adolescents require rates at the lower end.

### The Tri-Parameter Framework: Operationalizing Precision Fluid Management

**Figure 3.**
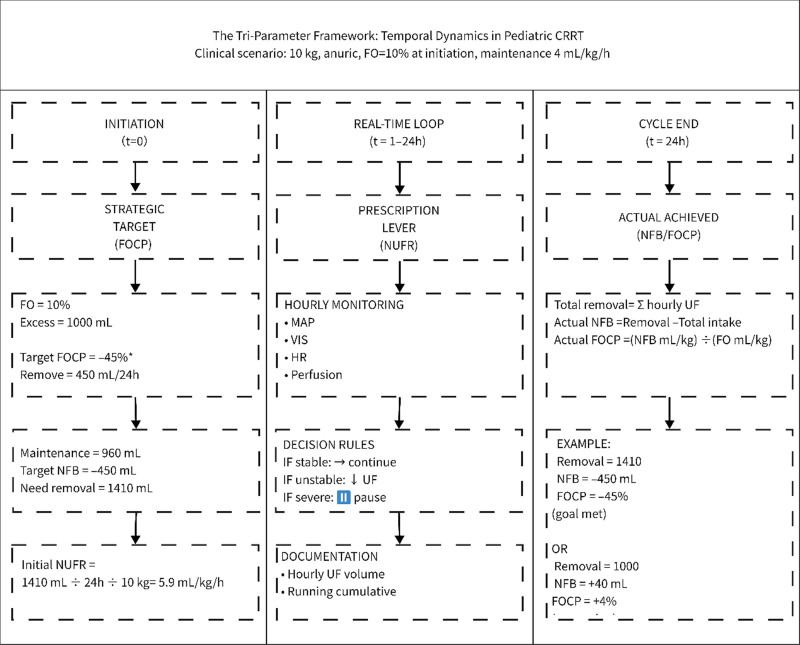
The tri-parameter framework: temporal dynamics in pediatric continuous renal replacement therapy (CRRT). This flowchart illustrates the distinct temporal roles of fluid overload clearance percentage (FOCP), net ultrafiltration rate (NUFR), and net fluid balance (NFB) in a dynamic clinical workflow. The example shows a 10 kg anuric child with 10% fluid overload (FO) at CRRT initiation, receiving maintenance fluids at 4 mL/kg/hr. At initiation (*t* = 0), FOCP serves as a strategic planning tool; the –45% target shown is the median from this study’s survivor cohort, presented as an illustrative benchmark (actual targets should be individualized). This goal translates to an initial NUFR of 5.9 mL/kg/hr—a starting point, not a fixed order. Throughout the 24-hour period (real-time loop), NUFR is adjusted hourly based on hemodynamic monitoring (MAP, vasoactive-inotropic score [VIS], heart rate [HR], perfusion). Decision rules: if stable, continue; if unstable, reduce UF; if severe, pause. At cycle end (*t* = 24 hr), actual NFB and FOCP are calculated retrospectively. Examples show goal met (removal = 1410 mL, NFB = –450 mL, FOCP = –45%) and suboptimal outcome (removal = 1000 mL, NFB = +40 mL, FOCP = +4%). These values inform next-cycle targets based on residual FO.

Based on these findings, we propose a tri-parameter framework in which each metric has a distinct temporal and functional role (**Fig. 3**). At initiation, FOCP serves as a strategic planning tool. Using the survivor-derived benchmark of –44.2% over 24 hours, a proportional removal goal is set. For a child with 10% FO (1000 mL excess), targeting FOCP of –40% to –50% translates to removing 400–500 mL in the first 24 h—a tangible, individualized goal. This FOCP target is translated into an NFB goal and then converted into an initial NUFR prescription—a starting point, not a fixed order.

Throughout the 24-hour period, this initial NUFR is adjusted hourly based on continuous hemodynamic monitoring; intra-day adjustments are guided primarily by hemodynamic tolerance, and when obligatory fluid inputs (e.g., blood products, albumin) are required, the clinician can either reduce other concurrent fluid infusions or temporarily escalate NUFR to maintain the FOCP target; adjustments are not driven by FOCP itself during the cycle. This real-time titration is essential, as hemodynamic instability may necessitate temporary reduction or pausing of ultrafiltration regardless of the planned target. At cycle end, actual NFB and FOCP are calculated retrospectively, quantifying how well the initial plan was executed under real-world physiologic constraints and informing next-cycle targets based on residual FO. This dynamic, iterative workflow ensures that the framework remains adaptive to the patient’s evolving clinical status rather than imposing rigid, time-invariant targets.

## LIMITATIONS AND FUTURE DIRECTIONS

This study has several limitations. The retrospective, single-center design limits causal inference and generalizability, and the calculation of pre-CRRT fluid balance is susceptible to documentation inaccuracies. Additionally, our analysis was restricted to the first 24 hours of CRRT, which may not capture the full dynamics of fluid management over a longer course. We also acknowledge that a substantial proportion of patients did not remain on CRRT beyond 48–72 hours; however, the exclusion of these patients from longer-window analyses would have introduced survivor bias. The 24-hour window was therefore a pragmatic choice to ensure cohort inclusiveness, but it remains a snapshot rather than a trajectory.

Accordingly, we have initiated a separate study examining FOCP trajectories over the first 72 hours of CRRT to address dynamic dose-response relationships beyond the initial 24-hour window. This ongoing work aims to characterize how FOCP evolves over multiple 24-hour cycles and whether trajectory patterns differ between survivors and nonsurvivors. Ultimately, the clinical impact of this framework requires validation in prospective, multicenter studies and, ideally, a randomized controlled trial comparing goal-directed therapy using the tri-parameter framework against standard care.

## CONCLUSIONS

We propose a paradigm shift in pediatric CRRT fluid management, transitioning from uniform ultrafiltration targets toward a sophisticated, individualized strategy. By concurrently leveraging the dynamic prescription lever (NUFR), the essential safety outcome (NFB), and the individualized clearance target (FOCP), clinicians can tailor therapy to each patient’s physiology, pathology, and needs. This integrated, tri-parameter framework promises to enhance the precision, efficacy, and safety of care for the most vulnerable children.

## ACKNOWLEDGMENTS

We extend our sincere appreciation to the Big Data Center of Children’s Hospital of Chongqing Medical University for its significant contributions during the data collection phase.

In the preparation of this work, the authors used Deepseek exclusively to enhance readability and language. After using this tool, the authors reviewed and edited the content as needed and take full responsibility for the content of the publication.

## Supplementary Material


