# Supplementary material for: Beyond Net Ultrafiltration Rate: A Precision Fluid Management Paradigm Integrating Three Key Parameters for Pediatric Continuous Renal Replacement Therapy
Source: Crit Care Explor. 2026 May 25;8(6):e1424. doi: 10.1097/CCE.0000000000001424 (PMC13200924; doi:10.1097/CCE.0000000000001424)
Supplement: Supplementary file 1 [file cc9-8-e1424-s001.pdf]

Title page

Article Title: Beyond Net Ultrafiltration Rate: A Precision Fluid Management Paradigm Integrating Three Key Parameters for Pediatric Continuous Renal Replacement Therapy

Manuscript ID: CCE-D-25-00444R1

Table of Contents

Table S1: Hemodynamic Trajectory from CRRT Initiation to 24 Hours in Survivors, Stratified by Key Physiological Subgroups: **Page 2-3**

IRB Supplementary Document:

Supplementary Explanation on Waiver of Informed Consent (Original Chinese Version): **Page 4**

Supplementary Explanation on Waiver of Informed Consent (English Translation Version): **Page 5**

Table S1. Hemodynamic Trajectory from CRRT Initiation to 24 Hours in Survivors, Stratified by Key Physiological Subgroups

Variable	Subgroup	n	CRRT Initiation (0h)	CRRT 24 Hours (24h)	*P*-value⁺
**MAP (mmHg)**	FO <5%	155	82.0 (64.0 to 96.0)	80.0 (65.0 to 92.0)	0.225
	FO ≥5%	84	64.5 (53.3 to 75.0)	70.5 (58.3 to 80.0)	0.010*
	CLI <3.3	129	77.0 (61.0 to 92.5)	74.0 (59.5 to 90.0)	0.572
	CLI ≥3.3	110	70.5 (59.0 to 89.0)	75.5 (62.8 to 89.0)	0.196
	BW <10 kg	64	56.5 (49.0 to 71.2)	60.0 (50.5 to 74.8)	0.409
	BW 10~20 kg	71	74.0 (61.0 to 90.0)	71.0 (62.0 to 84.0)	0.419
	BW 20~30 kg	29	87.0 (68.0 to 99.0)	88.0 (74.0 to 96.5)	0.456
	BW 30~40 kg	37	84.0 (69.0 to 100.5)	84.0 (72.0 to 98.0)	0.461
	BW ≥40 kg	38	83.0 (71.8 to 96.0)	84.5 (75.0 to 93.8)	0.264
**VIS**	FO <5%	155	0.0 (0.0 to 2.0)	0.0 (0.0 to 5.0)	0.313
	FO ≥5%	84	9.0 (0.0 to 30.0)	10.0 (0.0 to 24.3)	0.521
	CLI <3.3	129	0.0 (0.0 to 6.5)	0.0 (0.0 to 8.0)	0.705
	CLI ≥3.3	110	0.0 (0.0 to 16.5)	0.0 (0.0 to 16.0)	0.993

Variable	Subgroup	n	CRRT Initiation (0h)	CRRT 24 Hours (24h)	*P*-value[+]
	BW <10 kg	64	4.5 (0.0 to 23.0)	5.0 (0.0 to 20.5)	0.416
	BW 10~20 kg	71	0.0 (0.0 to 8.0)	0.0 (0.0 to 10.0)	0.794
	BW 20~30 kg	29	0.0 (0.0 to 5.0)	0.0 (0.0 to 2.5)	0.109
	BW 30~40 kg	37	0.0 (0.0 to 20.5)	2.0 (0.0 to 15.0)	0.550
	BW ≥40 kg	38	0.0 (0.0 to 7.5)	0.0 (0.0 to 8.5)	0.814

Data are presented as median (interquartile range). P-values are derived from paired Wilcoxon signed-rank tests comparing values at CRRT initiation (0h) and after 24 hours (24h) within each subgroup. Mean arterial pressure (MAP) significantly improved after 24 hours of CRRT specifically in subgroups with higher baseline fluid overload (FO ≥5%) and capillary leak (CLI ≥5), suggesting enhanced perfusion following fluid removal in these vulnerable populations. The vasoactive-inotropic score (VIS) remained stable across all subgroups, confirming the hemodynamic tolerability of the CRRT strategy. Abbreviations: MAP, mean arterial pressure; VIS, vasoactive-inotropic score; FO, fluid overload; CLI, capillary leak index; BW, body weight.

Supplementary Explanation on Waiver of Informed Consent (Original Chinese Version)

关于豁免知情同意的补充说明

申请人姓名:白科

申请审批项目名称: 儿童连续性肾脏替代治疗期间超滤及液体管理单中心回顾性研究

伦理批件号：2023 伦审研第 503 号

申请日期: 2023.11.20

批准日期:2023. 11. 20

经重庆医科大学附属儿童医院医学研究伦理委员会 2023.11.20 审查，该项目为利用以往临床诊疗中获得的病历/生物标本进行的回顾性研究，没有给受试者带来不必要的风险，在承诺不泄露患者隐私信息的情况下同意豁免知情同意。

重庆医科大学附属儿童医院医学研究伦理委员会

2024 年 5 月 2[illegible] 日

Supplementary Explanation on Waiver of Informed Consent (English Translation Version)

Supplementary Explanation on Waiving Informed Consent

Applicant's Name: Bai Ke

Application Approval Project Name: A Single-Center Retrospective Study on Ultrafiltration and Fluid Management During Continuous Renal Replacement Therapy in Children

Ethical Approval Number: File No. 2023(503)

Application Date: November 20, 2023

Approval Date: November 20, 2023

After review by the Medical Research Ethics Committee of Children's Hospital Affiliated to Chongqing Medical University - on November 20, 2023, this project is a retrospective study using the medical records/biological specimens obtained from previous clinical diagnoses and treatments. It does not pose unnecessary risks to the subjects. With the commitment not to disclose patient privacy information, the informed consent requirement has been waived.

The Medical Research Ethics Committee of the Children's Hospital Affiliated to Chongqing Medical University

May23, 2024
